# Markerless Knee Joint Position Measurement Using Depth Data during Stair Walking

**DOI:** 10.3390/s17112698

**Published:** 2017-11-22

**Authors:** Ami Ogawa, Akira Mita, Ayanori Yorozu, Masaki Takahashi

**Affiliations:** 1 School of Science for Open and Environmental Systems, Graduate School of Science and Technology, Keio University, 3-14-1 Hiyoshi, Kohoku-ku, Yokohama 223-8522, Japan; 2 Department of System Design Engineering, Keio University, 3-14-1 Hiyoshi, Kohoku-ku, Yokohama 223-8522, Japan; mita@sd.keio.ac.jp (A.M.); takahashi@sd.keio.ac.jp (M.T.); 3 Graduate School of Science and Technology, Keio University, 3-14-1 Hiyoshi, Kohoku-ku, Yokohama 223-8522, Japan; ayanoriyorozu@keio.jp

**Keywords:** stair climbing, stair descending, knee joint position, gait measurement, depth data, skeleton tracking, markerless measurement, Kinect v2, VICON, 3D motion capture system

## Abstract

Climbing and descending stairs are demanding daily activities, and the monitoring of them may reveal the presence of musculoskeletal diseases at an early stage. A markerless system is needed to monitor such stair walking activity without mentally or physically disturbing the subject. Microsoft Kinect v2 has been used for gait monitoring, as it provides a markerless skeleton tracking function. However, few studies have used this device for stair walking monitoring, and the accuracy of its skeleton tracking function during stair walking has not been evaluated. Moreover, skeleton tracking is not likely to be suitable for estimating body joints during stair walking, as the form of the body is different from what it is when it walks on level surfaces. In this study, a new method of estimating the 3D position of the knee joint was devised that uses the depth data of Kinect v2. The accuracy of this method was compared with that of the skeleton tracking function of Kinect v2 by simultaneously measuring subjects with a 3D motion capture system. The depth data method was found to be more accurate than skeleton tracking. The mean error of the 3D Euclidian distance of the depth data method was 43.2 ± 27.5 mm, while that of the skeleton tracking was 50.4 ± 23.9 mm. This method indicates the possibility of stair walking monitoring for the early discovery of musculoskeletal diseases.

## 1. Introduction

Due to a significant increase in the number of elderly people aged 65 years and older [1], there is a growing need for a method to find musculoskeletal diseases at an early stage. Stair walking monitoring is considered to be one of the solutions to meet this need. Subtle signs of musculoskeletal diseases are likely to be discovered earlier in tasks involving walking up and down stairs, because ascending and descending stairs are demanding activities [2,3,4]. In the case of knee osteoarthritis (OA), for example, which is a musculoskeletal disease that raises the risk of death [5], knee pain occurs in such stair walking activities before it does in other activities [6]. In addition, compared with controls, patients with knee OA show higher external knee adduction moments during stair walking [7,8], which contributes to the development of chronic knee pain [9].

However, it is difficult to recognize the gradual changes of musculoskeletal diseases by themselves. People do not usually go to see a doctor or monitor their musculoskeletal functions until the diseases have clearly developed. Furthermore, even if they have gait checks made in hospital, their performance in walking tests is not always the same as what it would be in a real living environment [10]. Stair walking monitoring in daily living environments without any disturbance is what is needed to screen people for clinical treatment.

A markerless method should be used in order not to mentally or physically disturb the subject. Previous studies have used 3D motion capture systems [11,12,13,14,15,16,17,18,19], electromyography (EMG) measurement devices [13,20,21], and electro-goniometers [20] to monitor stair walking. It is necessary to attach reflective markers or electrodes to the subject’s body so that these devices can record data. This makes these devices impractical for everyday living environments. Markerless sensors such as force plates [4,16,19,20,21] are widely used, but they are expensive and difficult to install. Video cameras must be installed on the side walls of stairs [2], but many households do not have stairwells wide enough. In addition, the use of cameras is best avoided owing to privacy concerns.

Depth sensors overcome many of the shortcomings of these other devices. They can be placed so that they face the subject’s frontal plane, and markers or electrodes are unnecessary, so installation is convenient. Skeleton tracking, which is a function provided by Kinect for Windows [22], makes it possible to perform a markerless joint position estimation based on machine learning using a number of datasets. Kinect can acquire kinematic and spatiotemporal parameters, and it has been used for level-walking performance evaluations [14,18]. 

**Figure 1:**
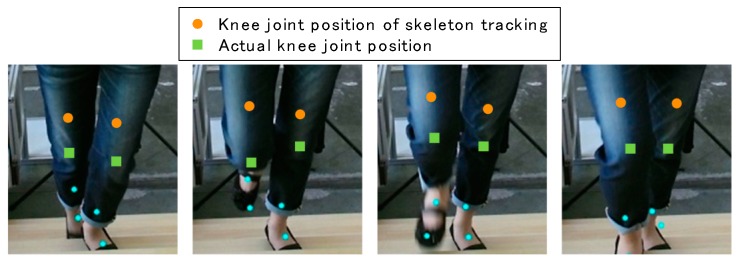
Knee joint position estimation during stair climbing. The knee joint positions detected by the skeleton tracking of Kinect v2 (orange circles) differ significantly from the actual ones (green squares).

As for stair walking monitoring, on the other hand, skeleton tracking has problems. In our previous study, we found empirically that the skeleton tracking of Kinect v2, the second generation of Kinect, is not good at estimating the knee joint positions during stair climbing [23]. Differences between actual knee joint positions and ones estimated by skeleton tracking are shown in Figure 1. The large differences suggest that a new method of stair walking monitoring should be developed.

The goal of our study is to develop a markerless method of stair walking monitoring. The present article reports (1) a new markerless method of knee joint position estimation by body range definition and leg data extraction that uses depth data acquired by Kinect v2, (2), an investigation on knee joint position estimation using the skeleton tracking function of Kinect v2, and (3), a comparison of the applicability of our method and skeleton tracking. A stair walking experiment was carried out with a precise 3D motion capture system.

This paper proceeds as follows. A description of our method is presented in Section 2. Experimental results are described in Section 3, and a discussion of the results and the validity of our method is presented in Section 4. Section 5 concludes the paper.

## 2. Materials and Methods

### 2.1. Definition and System Flow

We decided to use Kinect v2 for Windows (Microsoft, Redmond, WA, USA), which is an affordable and sufficiently high-quality depth sensor [24]. The joint positions of the skeleton tracking feature of Kinect v2 do not correspond exactly to the anatomical joint centers. They express positions on the surface of the joint because their values are based on depth data. Moreover, our method is based on depth data, so we aimed to calculate the knee joint position on the surface in the same way as the skeleton tracking of Kinect v2. Although our target is not the anatomical knee joint center, we use the term ‘knee joint position’. For ease of comparison, we put the reflective markers of the 3D motion capture system on the corresponding Kinect v2 knee joint positions, thus avoiding the need for conversion to the anatomical joint center.

**Figure 2:**
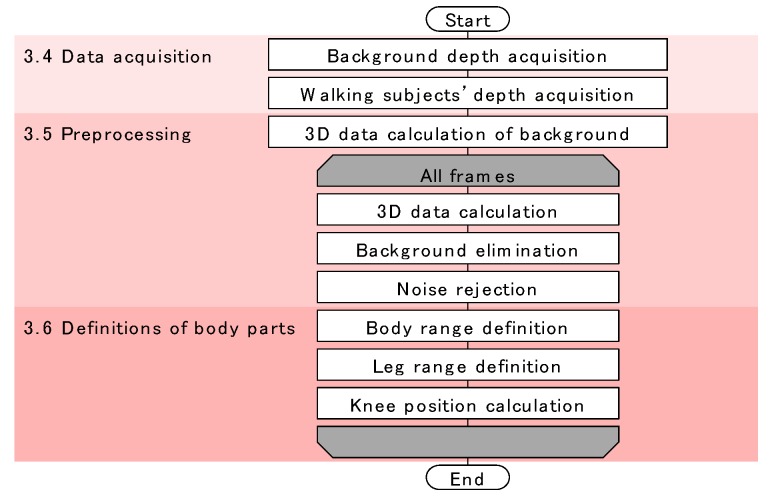
Flow of our system.

The flow of our method is shown in Figure 2. The method consists of three phases: data acquisition, preprocessing, and definition of body parts. The data acquisition phase was programmed in C++, and the other two phases were programmed in MATLAB 2017a (MathWorks Inc., Natick, MA, USA). These phases are completely independent and offline.

### 2.2. Kinect for Windows v2 by Microsoft

**Figure 3:**
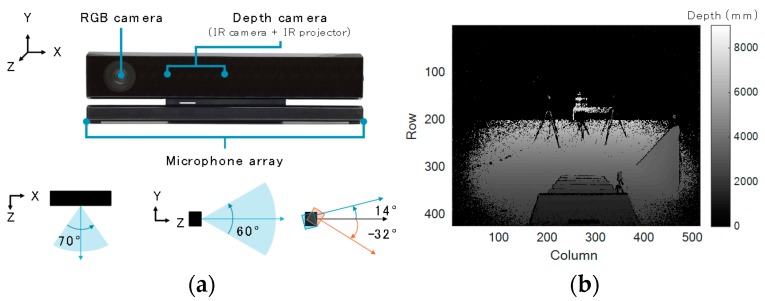
(**a**) Kinect v2 and its camera positions; (**b**) depth data acquired by Kinect v2.

Kinect v2 is composed of an RGB camera, a depth camera, and microphone array (Figure 3a). It acquires RGB data, depth data, and IR data at 30 fps. It also has a skeleton tracking function and face tracking function. Its horizontal and vertical visual angles are 70° and 60°, respectively. Depth data can be obtained in the range of 500 to 8000 mm, while the skeleton tracking data can be acquired in the range of 500 to 4500 mm. Although Kinect v2 has no tilt motor, it can be tilted from −32 to 14°. The accuracy of the depth data acquired by Kinect v2 was evaluated [25]. It was found that the error was less than 4 mm in an elliptical area, with a 3.5 m major axis around the subject. We acquired depth data (Figure 3b) for our method and skeleton tracking data for comparison. As our system is supposed to be used in homes, depth data is more suitable than RGB data in terms of privacy. The dimensions of Kinect v2 are defined as follows: mediolateral = *X*, vertical = *Y*, and anteroposterior = *Z*. We will simply refer to “Kinect v2” as “Kinect” in what follows.

### 2.3. Experimental Setup and Subjects

**Figure 4:**
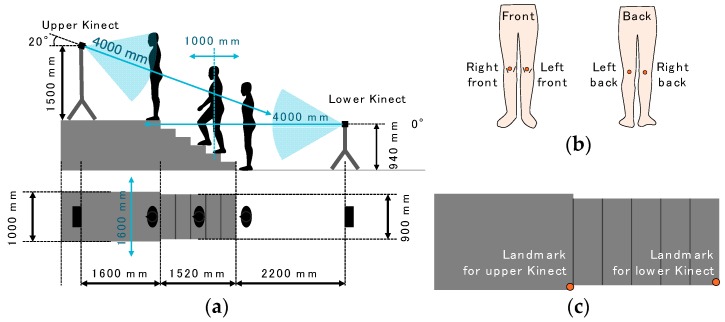
(**a**) Experimental setup; (**b**) positions of markers on a subject; (**c**) positions of markers on stairs as landmarks.

Our proposed method was compared with skeleton tracking of Kinect v2. Their results were evaluated with data collected by a 3D motion capture system (gold standard) that had been used in numerous previous studies [11,12,13,14,15,16,17,18,19]. The experiment was conducted at the Multi Media Room at Keio University in September 2015. The Multi Media Room is large enough to place a stage, stairs, two Kinects with tripods, and seven cameras with tripods of the 3D motion capture system. The setup is illustrated in Figure 4a. The staircase had six steps and a 1000-mm-high stage. The size of the stage was (W) 1000 mm × (D) 2000 mm × (H) 1000 mm. The width of the stairs was 900 mm, the run length was 304 mm, the riser height was 166 mm, and stair angle was 28.6°. The difference in height between the stage and the top of the stairs was 170 mm. 

Although stair climbing has been observed from the side of the subject in most previous studies, Kinects had to be set at the front or the back in our study, because our system is to be used in living environments. To determine an appropriate position, two Kinects were used; the lower one was set at 940 mm above the ground and 2200 mm away from the stairs; the upper one was set at 1500 mm above the stage and 1600 mm away from the stairs. The tilt of the upper one was −20°, and the tilt of the lower one was 0°. The tilts and positions of the Kinects were determined so that the whole body of each subject was within the devices’ visual range. Also, the distance from each Kinect to the subject was within 4 m because of the limitation of the skeleton tracking function.

For the use of the 3D motion capture system, four reflective markers were attached to the front and the back of the knees of each subject (Figure 4b), and each subject wore easily fitting pants of motion capture suits to reduce the influence of the clothes’ texture on the depth data. We recorded the 3D positions of the reflective markers at 200 Hz by using seven cameras (Bonita B10 by VICON [26]) set around the walking course. The beginning of both Kinects’ data acquisition was temporally synchronized with a voltage level change recorded by the VICON system, so the time counters of the two Kinects and VICON were synchronized. We used reflective markers of VICON as landmarks (Figure 4c) for alignment of each Kinect and VICON. The coordinates of the landmarks were subtracted from the data collected by the upper and lower Kinects.

Eight healthy students of Keio University, whose information is shown in Table 1, volunteered for the experiment. They provided informed consent. Each subject ascended and descended the stairs three times. The total number of ascents and descents for each subject was six. Though previous studies had considered the effect of the speed of approach to the stairs [27,28], the purpose of this experiment was to examine the accuracy of our method. Thus, subjects started to ascend and descend the stairs from a standing position with a self-selected comfortable speed.

### 2.4. Data Acquired by Kinect

Kinect provides depth data in the form of a 424 by 512 matrix per frame. Each cell of the matrix has the distance from Kinect to the object. Figure 3b shows an example of a depth image acquired by Kinect. The depth data in each cell is expressed as the color of the pixel.

The depth data was recorded every frame while the subject ascended and descended the stairs. Also, a clock program saved the time at which every depth data was collected. All these data were stored in memory once and written out as CSV files when the subject finished the stair walking test. The depth data of the background, which was used for the background elimination phase, was acquired before the stair walking test when no one was around the stairs. Figure 3b is a depth image of the acquired background depth data.

### 2.5. Preprocessing

#### 2.5.1. Three Dimensional Data Calculation and Tilt Correction

**Figure 5:**
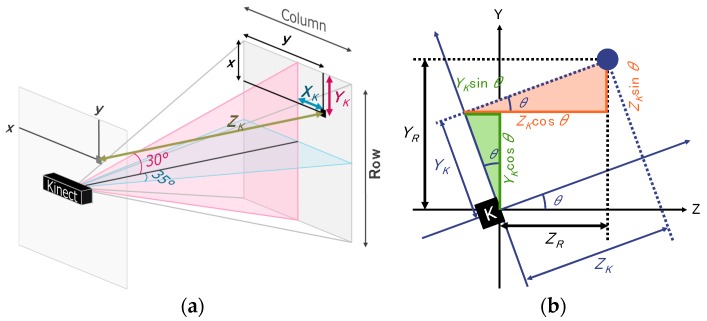
(**a**) Three-dimensional data calculation; (**b**) tilt correction.

The **X** matrix and **Y** matrix were calculated from the acquired depth data, i.e., the **Z** matrix, and the visual angle of Kinect, as follows:

(1) $$X_{K}\left(x,y\right)=\frac{\left(\left(col/2-y+1\right)\times Z_{K}\left(x,y\right)\times \tan\alpha \right)}{col/2}$$

(2) $$Y_{K}\left(x,y\right)=\frac{\left(\left(row/2-x+1\right)\times Z_{K}\left(x,y\right)\times \tan\beta \right)}{row/2},\;$$

where col=512, α=35°, and row=424, β=30°. The definitions of $X_{K}$, $Y_{K}$, and $Z_{K}$ are shown in Figure 5a.

When the Kinect was tilted, a tilt correction had to be made to the **Z** and **Y** matrices. The method, shown in Figure 5b, used Equations (3) and (4).

(3) $$Y_{R}\left(x,y\right)=Z_{K}\left(x,y\right)\times \sin\theta +Y_{K}\left(x,y\right)\times \cos\theta$$

(4) $$Z_{R}\left(x,y\right)=Z_{K}\left(x,y\right)\times \cos\theta -Y_{K}\left(x,y\right)\times \sin\theta ,\;$$

where θ means the tilt angle of the sensor. The preprocessing was applied to both the background data and each frame of data.

#### 2.5.2. Background Elimination and Noise Rejection

**Figure 6:**
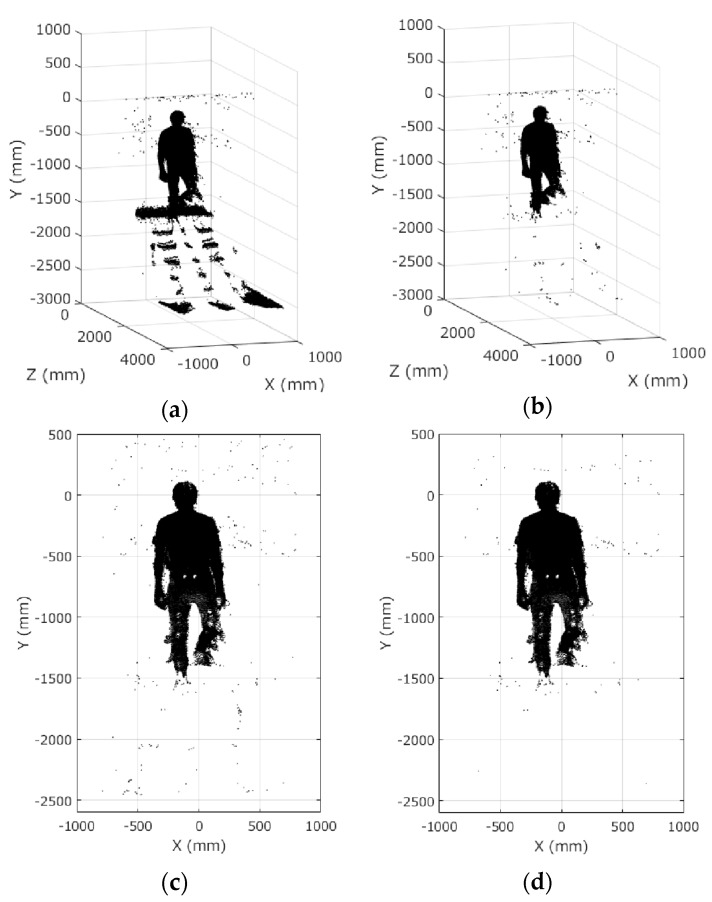
Background elimination and noise rejection: (**a**) before background elimination; (**b**) after background elimination; (**c**) after background elimination and before noise rejection; (**d**) after noise rejection.

The **X**, **Y**, and **Z** matrices of the background data were subtracted from those of each frame data. Then the 3D Euclidean distances of each cell were calculated. The cells, of which the 3D Euclidean distances were less than 50 mm, were regarded as background. The background cells in the **X**, **Y**, and **Z** matrices of each frame data were assigned zero. Figure 6a,b shows examples before and after the background elimination.

Zero was assigned to the appropriate cells of **X**, **Y**, and **Z** for the ranges in Equations (5)–(7). These ranges were determined according to the positions of the stairs and Kinect shown in Figure 4a.

(5) Z (x, y)> 4000

(6) X (x, y) < −800

(7) X (x, y) > 800

After that, the histogram of the **Z** matrix was calculated. The range that had the largest frequency except 0 was defined as the subject’s position on the *Z* axis. All data points that were farther than 500 mm from the subject’s position were deleted for noise rejection. Figure 6c shows the same data as Figure 6b after background elimination and before noise rejection. Figure 6d shows the result after noise rejection.

### 2.6. Definitions of Body Parts

#### 2.6.1. Body Range Definition and Leg Data Extraction

**Figure 7:**
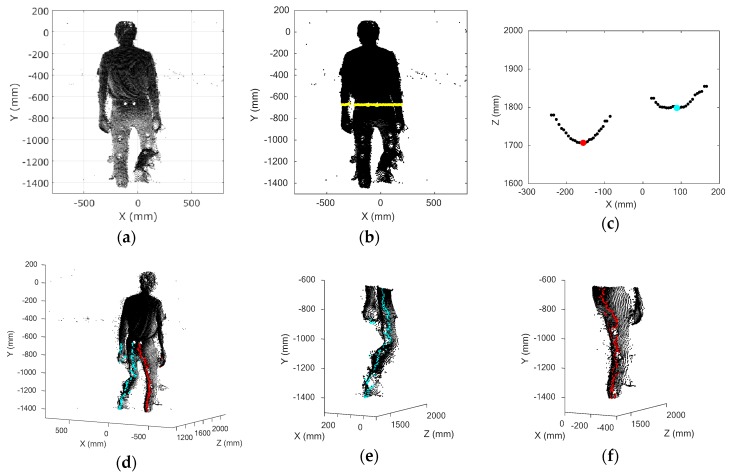
Body range definition and leg data extraction: (**a**) body extraction by using a histogram of *Y* values; (**b**) body plots and the boundary (yellow line) separating the body into upper and lower parts; (**c**) example of *X*-*Z* section at an arbitrary *Y* position; (**d**) representative positions of left and right legs; (**e**) separated left leg and its representative positions; (**f**) separated right leg and its representative positions.

The histogram of the **Y** matrix was calculated for the purpose of extracting the body from the recorded data. The bins with more than a frequency of 50 were used as the range of the body. The frequency threshold of 50 was very small, although the bin width was set automatically. For example, in the case of the lowest density of plots, when the smallest person (1540 mm tall) among the participants was at the farthest position from the Kinect, the *Z* axis distance was about 3300 mm and the body consisted of about 3900 plots. In this case, the maximum frequency was about 400, and the bin width was 100 mm. The cut-off of 50 worked as noise rejection for the *Y* axis. Among the extracted bins, the largest *Y* value was defined as the start position of the subject’s body, and the smallest *Y* value was defined as the end position of the subject’s body along the *Y* axis. Figure 7a shows the extracted 3D plots of the subject’s body.

The leg range was defined as the lower half of the height; 50% of the height from the bottom on the *Y* axis was defined as the boundary dividing the body into upper and lower parts. The sitting height ratio (SHR), which is calculated as (sitting height/stature)×100, is commonly used for body proportion evaluations [29]. According to a worldwide study on body proportion, the mean SHR of adults in a nation varies from 47.3 to 55.8 [30]. Thus, 50% is justified as a percentage of leg length to stature. The yellow line in Figure 7b expresses the defined boundary for the SHR of 50%. The plots below the boundary were taken to be the leg area.

Two clusters appear in the *X*-*Z* section in the leg area. An example in an arbitrary *X*-*Z* section is shown in Figure 7c. In this figure, the clusters form a curve, because the leg is in the shape of an ellipse. Thus, we defined the peak plots on the *Z* axis of two clusters as the representative positions of the left and the right legs in the *X*-*Z* section. The representative positions of both legs were defined all along the positions on the *Y* axis (Figure 7d). As the surface of the subject’s body was not so smooth, there were actually more than two peaks in the *X*-*Z* section, and it was not easy to determine the optimal representative positions of each leg. Therefore, we selected two peaks that had the largest peak width and set a rule that the peaks had to be separated by more than each subject’s ankle width (the thinnest leg area) to determine the optimal representative positions of each leg.

The boundary position on the *X* axis was calculated as the average of all representative positions of the left and right legs. The plots were separated into left and right legs by the position of the boundary on the *X* axis. The representative positions in each *X*-*Z* section were re-determined by finding the minimum plots (Figure 7e,f).

#### 2.6.2. Knee Joint Position Calculation

**Figure 8:**
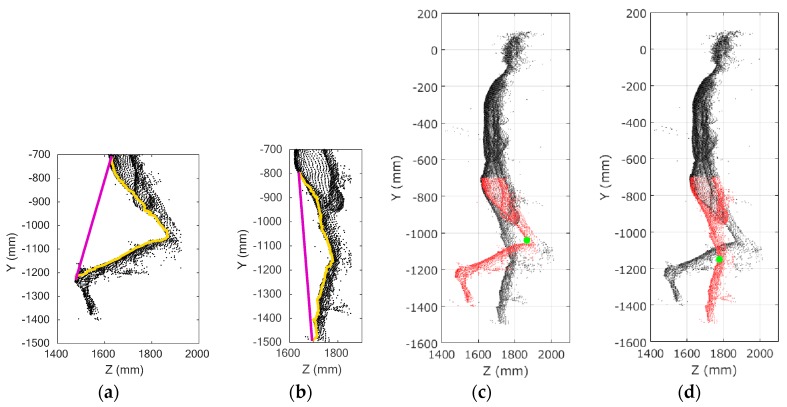
Knee joint position extraction: (**a**) plots of the left leg with representative positions (yellow line) and line connecting the top and bottom of the representative positions (purple line); (**b**) plots of the right leg with representative positions (yellow line) and line connecting the top and bottom of the representative positions (purple line); (**c**) plots of the left leg area (red dots) and estimated knee joint position of the left leg (green dot); (**d**) plots of the right leg area (red dots) and estimated knee joint position of the right leg (green dot).

The knee joint position was defined as follows. Figure 8a,b show plots of the left and right legs in the *Z*-*Y* plane. The yellow lines in the figures express representative positions of the left and right legs at each height, and the purple lines connect the top and bottom representative positions. The knee joint position was defined as the point on the yellow line that had the largest distance from this purple line. Figure 8c,d shows the estimated knee joint positions as green dots and the leg areas as red dots.

There was a problem that the knee joint position may be possibly falsely estimated when the leg is extended. Therefore, whether the leg was extended or not was checked after the knee joint position was estimated once. The leg was considered to be extended if the distance from the estimated knee joint position to the purple line was less than the threshold. The threshold was determined to be 100 mm after examining the subjects’ data. In the case of leg extension, the distance between the estimated knee joint position and the latest knee joint position was calculated. If the distance was larger than the threshold, the leg area was reduced to an area covering a distance of 100 mm on the *Y* axis from the latest knee joint position. Then the purple and yellow lines were drawn again, and the knee joint position was estimated. By extracting the leg area around the correct knee joint position, the risk of misrecognition could be reduced. The threshold was determined to be 150 mm by considering the sampling time and displacement of the knee joint. This threshold was also used for the abnormal data on the knee joint position, as described in Section 2.7. In this case, the range of 100 mm was determined through consideration of the frame rate and the walking speed. This method used the latest knee joint position, and that is why it was applied only after the knee joint position was acquired once.

### 2.7. Data Analysis

We obtained the depth data and skeleton tracking data of the subjects from Kinect at about 30 frames per second and the 3D position data of four markers from the VICON system at 200 frames per second. We estimated the 3D knee joint positions using the proposed method. To calculate the error for each point of Kinect data, the data of VICON nearest to that time were selected. In order to avoid the errors caused by the interpolation for upsampling, the VICON data were downsampled to the same number of frames as that of Kinect. Data points that showed sudden changes (>150 mm) from the previous data points were rejected as abnormal.

The present method was evaluated using Pearson’s correlation coefficients (*r*), 95% confidence intervals of the intraclass correlation coefficient (ICC) case 2 (ICC (2,1)), signal-to-noise ratio (SNR), and the 3D Euclidian distance (3D error). The front (back) knee joint position was estimated from depth data captured by the upper (lower) Kinect device during stair ascents. The front (back) knee joint position was estimated from the depth data captured by the lower (upper) Kinect device during stair descents. All analyses were applied to compare our method with VICON and to compare the Kinect skeleton tracking with VICON. The *r* and SNR values were applied to every frame of each trial of each subject. On average, 230 frames (samples) were collected for each trial. Based on the calculation of G*Power 3 1.9.2 (Universität Düsseldorf, Düsseldorf, Germany), the appropriate sample size for correlation analysis was 82. Thus, the sample size was enough.

Values for *r* were calculated using MATLAB 2017a to assess the linear relationship between the knee joint positions acquired by two methods. The ICC (2,1), which expresses the Inter-rater reliability, was used to evaluate the agreement between the results obtained by the two measurement systems. This was calculated using IBM SPSS Statistics, version 24 (IBM, Armonk, NY, USA). We used SNRs based on the variance of the signals to quantify the noise relative to the VICON data, as described previously [18]. The SNRs, defined in Equations (8)–(10), included the usual transformation into decibels [31].

(8) $${\mathrm{SNR}}_{x}=\;20\log_{10}\left(\frac{variance\left(X_{VICON}\right)}{variance\left(X_{error}\right)}\right),$$

(9) $${\mathrm{SNR}}_{y}=\;20\log_{10}\left(\frac{variance\left(Y_{VICON}\right)}{variance\left(Y_{error}\right)}\right),$$

(10) $${\mathrm{SNR}}_{z}=\;20\log_{10}\left(\frac{variance\left(Z_{VICON}\right)}{variance\left(Z_{error}\right)}\right),$$

where *X_error_*, *Y_error_*, and *Z_error_* are the errors of the values estimated by our method compared with the VICON values:

(11) $$X_{error}=\;\left|X_{Proposed}-X_{VICON}\right|$$

(12) $$Y_{error}=\;\left|Y_{Proposed}-Y_{VICON}\right|$$

(13) $$Z_{error}=\;\left|Z_{Proposed}-Z_{VICON}\right|$$

The 3D error between the estimated position and the marker position of VICON was calculated as follows:

(14) $$3Derror=\;\sqrt{X_{error}{}^{2}+Y_{error}{}^{2}+Z_{error}{}^{2}}.$$

## 3. Results

As shown in Table 2, all results of *r*_y_ and *r*_z_ are larger than 0.9. This indicates a strong correlation, which is defined as an ‘excellent relationship’ [18,32] between our method and the VICON measurement in the case of the *Y* and *Z* axes position measurement. In the case of *X* axis position measurement, on the other hand, the values of *r*_x_ are so small that our method and the VICON measurement are in a ‘poor’ or ‘moderate relationship’ [18,32]. The tendency of the SNRs is similar to the values of *r*. The values are assessed as follows [18]: SNR < −20 dB means that the data are ‘altered or influenced by large noise’; −20 dB < SNR < 20 dB means that they are ‘often influenced by small noise or small systematic bias’; and SNR > 20 dB means that they are ‘accurate enough’. According to Table 2, SNRy and SNRz indicate the data of *Y* and *Z* axes are ‘accurate enough’, but SNRx indicates that the data of *X* axis are ‘influenced by small noise’. The ICC (2,1) values are over 0.800 for the *X* axis and over 0.900 for the *Y* and *Z* axes; these are considered ‘good’ and ‘excellent reliability’, respectively [33]. The results for the Kinect skeleton tracking showed a similar tendency to those for our method.

**Figure 9:**
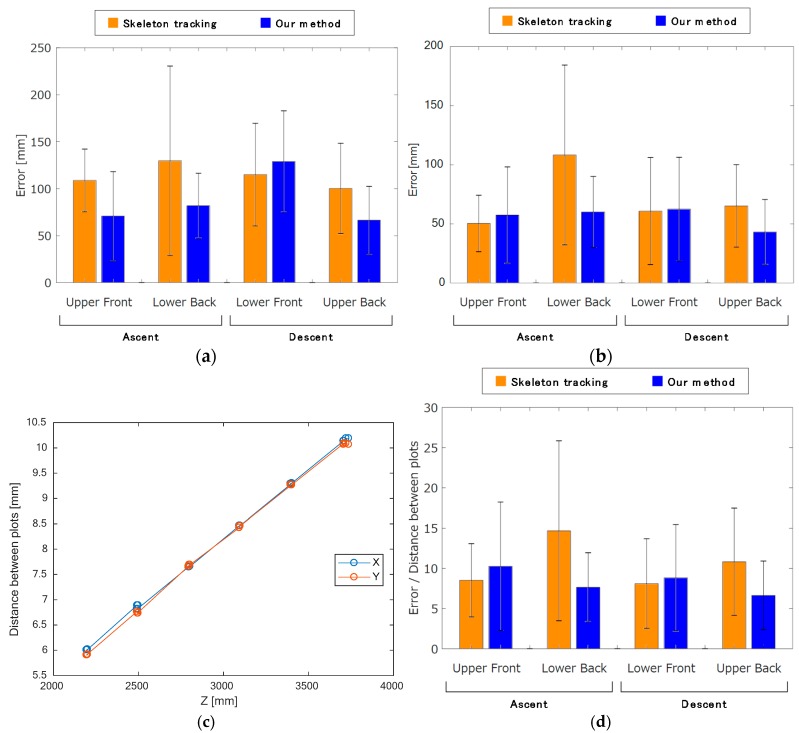
3D error of our method compared with the Kinect skeleton tracking method: (**a**) unadjusted 3D error; (**b**) zero-mean shifted 3D error; (**c**) relation between resolution and distance from the Kinect sensor; (**d**) normalized 3D error. Data are shown as mean ± standard deviation.

The results of our method are shown in Figure 9a together with those of the Kinect skeleton tracking for comparison. For all conditions, the 3D errors for our method, except that of the third condition (lower Kinect, descent captured from the front), were smaller than those for the Kinect skeleton tracking. This means that our method has a smaller bias error than skeleton tracking, except for the third condition.

To eliminate the bias error, the mean value of the data for each dimension was subtracted from the data of each dimension, for example:

(15) $$zeromeanX_{Proposed}=\;X_{Proposed}-\mathrm{mean}\left(X_{Proposed}\right),$$

(16) $$zeromeanX_{VICON}=\;X_{VICON}-\mathrm{mean}\left(X_{VICON}\right).$$

Figure 9b shows the zero-mean shifted 3D errors for our method, together with those for skeleton tracking. Compared with Figure 9a, it can be seen that the zero-mean shifted error for the third condition (lower Kinect, descent captured from the front) of our method is significantly improved from the un-shifted data, as there was a large bias error. The skeleton tracking results also had bias errors.

The distance from the Kinect affects the resolution of the data. To normalize the results to mitigate this issue, we used the following process. Four plots were chosen per six risers to examine the resolution of Kinect at various distances. The distance and the number of plots between pairs of selected plots on the *X* and *Y* axes were acquired. We calculated the resolution for each axis as the distance between the two selected plots divided by the number of plots. Figure 9c shows that there is a linear relationship between the distance and the number of plots, and the data for both axes correlate well. These relations are expressed below:

(17) $$Resolution_{x}=\;0.0027\times Distance-0.0265$$

(18) $$Resolution_{y}=\;0.0028\times Distance-0.1238$$

To consider the effect of resolution, the 3D error of each frame was divided by its resolution, as calculated using the equation above. Equation (17) was used for normalizing the 3D error because the distances of the *X* axis were larger than those of the *Y* axis. Figure 9d shows that the normalized 3D error of our method for both ascent and descent was the smallest when the data were captured from the back. Conversely, the skeleton tracking method showed better results when the data were measured from the front compared with from the back.

## 4. Discussion

### 4.1. Accuracy Comparison and Applications of Our Method

All subjects’ data were certainly acquired, and our method calculated the subject’s knee joint positions during stair walking from Kinect’s depth data. Since our system works without any restrictions on the subject, it can be installed in houses and used for daily stair walking measurements. The most remarkable thing is that it can be used by non-professionals. The system is simple to set up: the only hardware is a Kinect on a tripod connected to a PC. Our system may be able to be used to screen subjects for abnormal gait on stairs, and it may enable doctors to monitor the rehabilitation of patients after they have been discharged from a hospital by installing it in the patients’ houses.

From Figure 9d, we conclude that our method more accurately estimates the knee joint position than skeleton tracking during stair walking tasks. Kinect is normally set up facing the target subject, as it was originally developed as a game controller. Thus, even when the subject turns his back on the Kinect, the skeleton tracking recognizes the subject as if s/he is facing to it. This fact is probably why the results of skeleton tracking were better when the data was captured from the front than from the back.

From these results and those of the previous study shown in Figure 1, it is clear that the accuracy of skeleton tracking depends on the tilt of the Kinect. The ratio of run to rise of a staircase has an influence on stair walking monitoring. It is important that Kinect can capture the subject’s whole body while it is less tilted. However, it is difficult to do so when the gradient of the staircase is steep like in Figure 1 (an angle of 35.6°). Accordingly, our method is likely to be more versatile than skeleton tracking.

A previous study indicated that a 3D Error of more than 0.05 m for the skeleton joint positions was a large error in several tasks [18]. That study used a clinical parameter and knee displacement in the evaluation of walking on the spot. Accuracy and reliability of all clinical analyses were high enough, while the accuracy (3D error) of the knee joint position was 0.04 ± 0.01 m. On the other hand, our method had accuracies of 60.1 ± 30.1 mm (lower Kinect, ascent captured from the back) and 43.2 ± 27.5 mm (upper Kinect, descent captured from the back). Although both of these values had larger errors than in the previous study; the *r* of our method were better than theirs on the *X*, *Y*, and *Z* axes. Therefore, we conclude that our method is accurate enough to estimate knee joint positions. 

The source of the ‘poor’ or ‘moderate relationship’ with the reference values in *r*_x_ can be explained by the small movement of the knee joint on the *X* axis. Although they are not written in order to avoid the redundancy, the orders of magnitude of absolute errors in each axis were the same. Thus, the correlation of the 3D positions of two methods can be considered ‘good’.

The ability of our method to acquire the actual clinical parameters should be confirmed in order to clarify its usefulness. In addition, we tested our method on only young healthy subjects. Additional experiments should be conducted with elderly persons and patients with musculoskeletal diseases. Moreover, our experiment used a six step staircase, and the maximum distance from the subjects to the Kinect was about 4 m. That means our results only cover the stair walking task in area range up to 4 m from the Kinect. Its usability on other staircases, which have different ratios of runs to rises, should also be examined.

### 4.2. Descent

**Figure 10:**
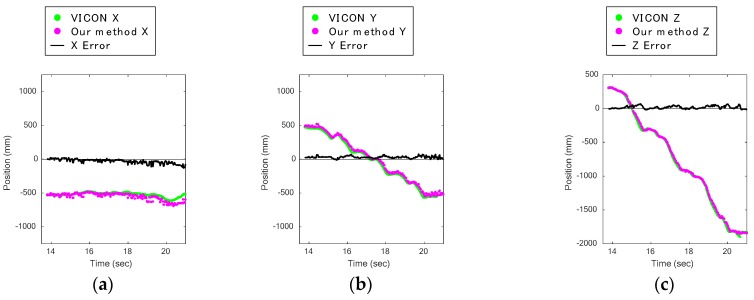

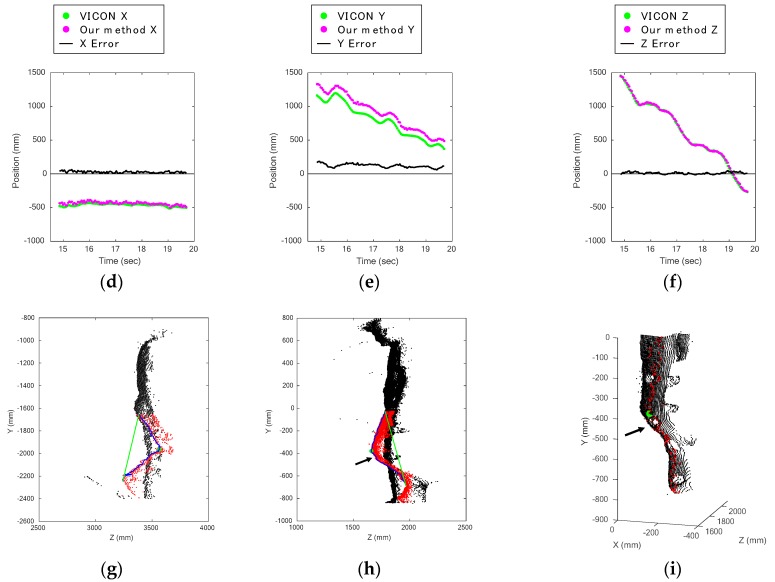
Front silhouette around the knee is round during descent: (**a**) knee displacement on *X* axis during stair descent captured by upper Kinect; (**b**) knee displacement on *Y* axis during stair descent captured by upper Kinect; (**c**) knee displacement on *Z* axis during stair descent captured by upper Kinect; (**d**) knee displacement on *X* axis during stair descent captured by lower Kinect; (**e**) knee displacement on *Y* axis during stair descent captured by lower Kinect; (**f**) knee displacement on *Z* axis during stair descent captured by lower Kinect; (**g**) estimated knee joint position (green dot) and leg area (red dots) during descent captured by upper Kinect; (**h**) estimated knee joint position (green dot), leg area (red dots), and the position of reflective marker of VICON (data hole indicated by an arrow) during descent captured by lower Kinect; (**i**) 3D visualization of Figure 10g; estimated knee joint position (green dot) is located above the position of reflective marker of VICON (hole indicated by an arrow).

Figure 9a,b indicates that the lower Kinect had significantly poorer results and that its data had large bias errors. Figure 10a,c shows the knee displacements on the *X*, *Y*, and *Z* axes of a trial during a descent captured by the upper Kinect, and Figure 10d–f shows those captured by the lower Kinect. Comparing these figures, it can be seen that the plots of our method for the *Y* axis in Figure 10e of the lower Kinect are rather different. They are always higher by a certain amount than the VICON data.

Figure 10h shows subject 6’s descent in the *Z*-*Y* plane captured by the lower Kinect. For comparison, Figure 10g shows the same subject’s descent captured at the same time as Figure 10h but with the upper Kinect. The green dot shows the estimated knee joint position, and the red dots show the leg area. In Figure 10h, the silhouette of the knee area is round. The leg area plots in 3D of the same data as in Figure 10h are shown in Figure 10i. The arrows in Figure 10h,i point to the correct knee joint positions, where the reflective marker of VICON was put. The data of plots for the reflective markers could not be acquired, so their positions appear as holes in the data in Figure 10i. According to the two figures, it can be seen that the estimated knee joint position (the green dot) is located above the correct knee joint position. It is hard to find the correct knee joint positions by finding the positions that are farthest from the purple line in Figure 8a, because the front silhouette of the knee area is round.

The above results were compensated by subtracting a constant bias error, as described in Section 3. However, in Figure 9d, the results for descents captured from the back are better than those captured from the front. Hence, we conclude from these results that stair descents should be captured from the back. The results likely include not only the bias error but also other errors.

### 4.3. Ascent

**Figure 11:**
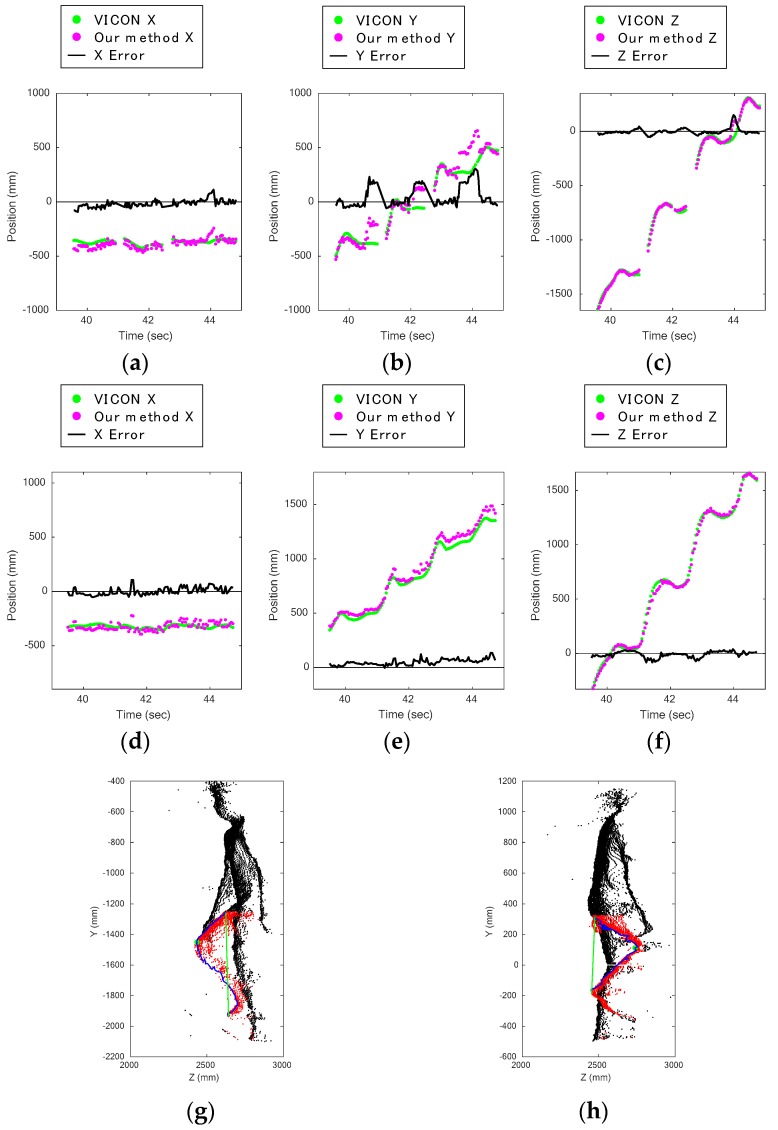
Comparison of results during ascent captured from the front and the back: (**a**) knee displacement on *X* axis during stair ascent captured by upper Kinect; (**b**) knee displacement on *Y* axis during stair ascent captured by upper Kinect; (**c**) knee displacement on *Z* axis during stair ascent captured by upper Kinect; (**d**) knee displacement on *X* axis during stair ascent captured by lower Kinect; (**e**) knee displacement on *Y* axis during stair ascent captured by lower Kinect; (**f**) knee displacement on *Z* axis during stair ascent captured by lower Kinect; (**g**) estimated knee joint position (green dot) and leg area (red dots) during ascent captured by upper Kinect; (**h**) estimated knee joint position (green dot) and leg area (red dots) during ascent captured by lower Kinect.

Figure 11a–c shows the knee displacement on the *X*, *Y*, and *Z* axes, as captured by the upper Kinect during stair ascents, and Figure 11d–f shows those captured by the lower Kinect. Comparing these figures, it is clear that the errors on the *Y* axis data captured by the upper Kinect are larger than the others (Figure 11b). One of the main error sources was that the method did not work well when the silhouettes around the knee were round. Figure 11g,h contains data of subject 8 climbing the stairs, which were captured at the same time from the front and back by the upper and lower Kinect. The data show that the back of the knee bent at a sharper angle than the front.

According to the heights of ascent in Figure 9b,d, we can see that the lower Kinect data was affected by the resolution, as the distance from the lower Kinect was longer than that of the upper Kinect. In other words, other disturbances more greatly affected the upper Kinect data, as is indicated by the larger standard deviation of the upper Kinect data. In light of the discussion in Section 4.2, we conclude that the stair walking should be captured from the back, not the front.

### 4.4. Possible Sources of Error

**Figure 12:**
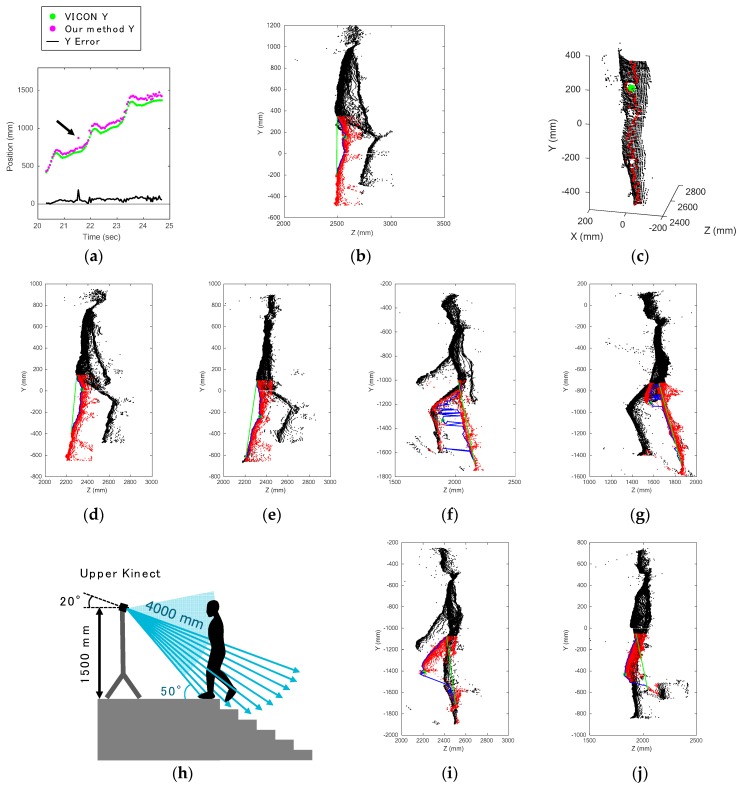
(**a**) Subject 8’s knee displacement on *Y* axis during third stair ascent (abnormal data indicated by an arrow); (**b**) estimated knee joint position (green dot) that was misrecognized and leg area (red dots); (**c**) a data hole made by a reflective marker on the thigh was falsely recognized as the knee joint position; (**d**) instance in which the boundary of the gluteus maximus and thigh was falsely recognized during ascent captured from the back in a male subject; (**e**) comparison of Figure 10d: the same situation of a female subject; (**f**) error caused by confusion of left and right leg data; (**g**) error caused by noise due to hands; (**h**) location giving the lowest density of plots of the shins in the case of the upper Kinect; (**i**) example of low-density plots of the shins during ascent captured by the upper Kinect; (**j**) example of low-density plots of the shins during descent captured by the lower Kinect.

We checked all errors that behaved differently from the others, such as the plots pointed to by the arrow in Figure 12a. Except for bias errors, there were few errors when the leg was bent. Accordingly, this method can accurately provide the knee joint positions when the knee joint angles are large.

The strongest error sources had to do with misrecognition of the thigh position. There were two sources. One was the round shape of the area around the knee, as we already mentioned in Section 4.2 and Section 4.3. The other was the influence of the reflective markers of VICON. The examples shown in Figure 12b,c correspond to the time indicated by the arrows in Figure 12a. From Figure 12c, it can be seen that the reflective marker on the thigh made the surface uneven, thereby causing a deficiency of data points, and it was falsely recognized as the knee joint position. The data points of the reflective markers could not be acquired; they appear as holes in Figure 10i and Figure 12c. 674 frames were falsely recognized for this reason: 444 frames of ascents and 134 frames of descents captured by the upper Kinect, and 96 frames of descents captured by the lower Kinect. Incidentally, misrecognition of the shin position happened in 34 frames during ascents captured by the upper Kinect.

The errors also depended on the subject’s bodily proportions and posture. Here, as an example of gender differences, the misrecognition due to the bulge of the gluteus maximus muscle happened in only the male subjects’ data. The boundary between the gluteus maximus and thigh was often falsely recognized as the knee joint position during ascents captured from the back. A misrecognized frame of subject 6, who is male, is shown in Figure 12d, and ascending data captured from the back of subject 10, who is female, is shown in Figure 12e. In this case, it can be said that the male subject had a more muscular build. When his leg was almost completely extended, the other uneven parts were very likely to be falsely recognized as knee joint positions. All such errors happened during ascents captured by the lower Kinect.

On the other hand, the left and right legs were relatively difficult to distinguish in the female subjects’ data, as the female subjects tended to position their legs closer together when they walked. Figure 12f shows an error caused by confusion of the left and right leg data. In this case, some of the left leg plots were misrecognized as right leg plots, and as the left leg was positioned ahead, the representative plots were defined incorrectly. In some frames, the left and right legs could not be separated by the vertical plane, so a more complex boundary plane should have been used.

The data of subjects who moved their hands in the front of their legs when climbing the stairs had errors. These errors only happened in the frames during ascents captured from the front by the upper Kinect. When the subject was climbing the stairs, the legs moved above and came closer to the arms. We speculate that this caused the errors. Figure 12g is an example of an error caused by a hand. The hands should have been rejected in the body range definition. Despite this, such noise was never observed in the data captured from the back, because the subjects did not move their arms behind, only ahead. Therefore, the hands do not have to be rejected when our system always captures the subject from the back. In other words, this fact reinforces the conclusion that stair walking should be observed from the back.

The density of the data plots became quite low when the subjects’ shins were parallel to the radial rays of the depth sensor. The case of the upper Kinect is shown in Figure 12h. In our study, this situation happened in frames which were captured from the front. Examples are shown in Figure 12i,j. Despite the low density of plots for shins, the knee joint position estimation worked well.

## 5. Conclusions

This study suggested that a markerless measurement system working by body range definition and leg data extraction can be used for estimating knee joint positions during stair walking activities. The accuracy of our method, which uses depth data from Kinect v2, and that of the skeleton tracking function of Kinect v2 were evaluated by using the data acquired by a 3D motion capture system as a reference. The experimental results indicate that our estimation method during stair walking is more accurate than the skeleton tracking of Kinect v2. They show that our system can estimate the knee joint positions within 43.2 ± 27.5 mm of 3D Euclidian distance errors, while Pearson’s correlation coefficients of the anteroposterior dimension (*Z* axis) and vertical dimension (*Y* axis) are over 0.9. This suggests that stair walking activity can be measured from the back, because there is less noise in the depth data captured from the back than from the front. In the future, an experiment will be carried out with more subjects, including the people who have recently developed musculoskeletal diseases, and variously designed staircases in an attempt to evaluate the practicality of the present knee joint position estimation method.

## Figures and Tables

**Table 1:** Characteristics of the subjects.

No	Sex	Age	Mass (kg)	Height (mm)	Inter-ASIS Distance (mm)	Leg Length (mm)	Knee Width (mm)	Ankle Width (mm)
1	F	24	48	1540	280	800	100	70
2	M	24	55	1730	290	910	115	70
3	M	24	80	1770	310	910	110	90
4	M	21	73	1800	260	960	110	80
5	F	24	43	1510	270	780	90	80
6	F	24	51	1625	300	840	110	75
7	F	25	44	1540	270	800	100	80
8	F	24	49	1580	250	830	100	75
Mean ± SD		24 ± 1	55 ± 13	1636 ± 107	279 ± 19	854 ± 61	104 ± 8	78 ± 6

**Table 2:** Accuracy of knee joint positions estimated by our method relative to VICON values: Pearson’s correlation coefficients and signal-to-noise ratio.

		Pearson’s Correlation Coefficients			Signal-to-Noise Ratio		
		*r*_x_	*r*_y_	*r*_z_	SNR_x_	SNR_y_	SNR_z_
Ascent	Upper Kinect, Front	0.561 (0.180)	0.983 (0.011)	0.998 (0.001)	−4.21 (4.57)	30.3 (5.91)	51.0 (5.72)
	Lower Kinect, Back	0.687 (0.162)	0.978 (0.022)	0.998 (0.002)	0.744 (7.05)	28.7 (6.23)	49.6 (6.96)
Descent	Lower Kinect, Front	0.339 (0.222)	0.994 (0.004)	0.996 (0.002)	−10.7 (5.14)	36.8 (4.11)	43.2 (5.73)
	Upper Kinect, Back	0.608 (0.223)	0.994 (0.007)	0.999 (0.001)	−5.47 (5.58)	44.6 (9.66)	54.6 (5.90)

## References

[1] National Institutes of Health (2011). Global health and aging. Natl. Inst. Health Publ..

[2] Bergmann G., Deuretzbacher G., Heller M., Graichen F., Rohlmann A., Strauss J., Duda G. (2001). Hip contact forces and gait patterns from routine activities. J. Biomech..

[3] Kutzner I., Heinlein B., Graichen F., Bender A., Rohlmann A., Halder A., Beier A., Bergmann G. (2010). Loading of the knee joint during activities of daily living measured in vivo in five subjects. J. Biomech..

[4] Liikavainio T., Isolehto J., Helminen H.J., Perttunen J., Lepola V., Kiviranta I., Arokoski J.P., Komi P.V. (2007). Loading and gait symmetry during level and stair walking in asymptomatic subjects with knee osteoarthritis: Importance of quadriceps femoris in reducing impact force during heel strike?. Knee.

[5] Nüesch E., Dieppe P., Reichenbach S., Williams S., Iff S., Jüni P. (2011). All cause and disease specific mortality in patients with knee or hip osteoarthritis: Population based cohort study. BMJ.

[6] Hensor E., Dube B., Kingsbury S.R., Tennant A., Conaghan P.G. (2015). Toward a Clinical Definition of Early Osteoarthritis: Onset of Patient-Reported Knee Pain Begins on Stairs. Data From the Osteoarthritis Initiative. Arthritis Care Res..

[7] Kenji T., Masaya A., Yoshio W., Hiroka H., Kazuki T., Makoto T., Kōichi S. (2015). Characteristics of External Knee Adduction Moment during Stair Descent and Level Walking: Comparison of Healthy Elderly People and Patients with Knee Osteoarthritis. Rigakuryoho Kagaku.

[8] Sacco I., Trombini-Souza F., Butugan M., Passaro A., Arnone A., Fuller R. (2012). Joint loading decreased by inexpensive and minimalist footwear in elderly women with knee osteoarthritis during stair descent. Arthritis Care Res..

[9] Amin S., Luepongsak N., McGibbon C.A., LaValley M.P., Krebs D.E., Felson D.T. (2004). Knee adduction moment and development of chronic knee pain in elders. Arthritis Care Res..

[10] Wang F., Stone E., Skubic M., Keller J.M., Abbott C., Rantz M. (2013). Toward a passive low-cost in-home gait assessment system for older adults. IEEE J. Biomed. Health Inform..

[11] Hicks-Little C.A., Peindl R.D., Fehring T.K., Odum S.M., Hubbard T.J., Cordova M.L. (2012). Temporal-spatial gait adaptations during stair ascent and descent in patients with knee osteoarthritis. J. Arthroplast..

[12] Hicks-Little C.A., Peindl R.D., Hubbard T.J., Scannell B.P., Springer B.D., Odum S.M., Fehring T.K., Cordova M.L. (2011). Lower extremity joint kinematics during stair climbing in knee osteoarthritis. Med. Sci. Sports Exerc..

[13] Hinman R.S., Bennell K.L., Metcalf B.R., Crossley K.M. (2002). Delayed onset of quadriceps activity and altered knee joint kinematics during stair stepping in individuals with knee osteoarthritis. Arch. Phys. Med. Rehabilit..

[14] Mentiplay B.F., Perraton L.G., Bower K.J., Pua Y.-H., McGaw R., Heywood S., Clark R.A. (2015). Gait assessment using the Microsoft Xbox One Kinect: Concurrent validity and inter-day reliability of spatiotemporal and kinematic variables. J. Biomech..

[15] Nicolas R., Nicolas B., François V., Michel T., Nathaly G. (2015). Comparison of knee kinematics between meniscal tear and normal control during a step-down task. Clin. Biomech..

[16] Novak A., Brouwer B. (2011). Sagittal and frontal lower limb joint moments during stair ascent and descent in young and older adults. Gait Posture.

[17] Novak A., Komisar V., Maki B., Fernie G.R. (2016). Age-related differences in dynamic balance control during stair descent and effect of varying step geometry. Appl. Ergon..

[18] Otte K., Kayser B., Mansow-Model S., Verrel J., Paul F., Brandt A.U., Schmitz-Hübsch T. (2016). Accuracy and reliability of the kinect version 2 for clinical measurement of motor function. PLoS ONE.

[19] Reid S.M., Graham R.B., Costigan P.A. (2010). Differentiation of young and older adult stair climbing gait using principal component analysis. Gait Posture.

[20] Hsu M.-J., Wei S.-H., Young-Hue Y., Ya-Ju C. (2007). Leg stiffness and electromyography of knee extensors/flexors: Comparison between older and younger adults during stair descent. J. Rehabilit. Res. Dev..

[21] Larsen A.H., Puggaard L., Hämäläinen U., Aagaard P. (2008). Comparison of ground reaction forces and antagonist muscle coactivation during stair walking with ageing. J. Electromyogr. Kinesiol..

[22] Shotton J., Sharp T., Kipman A., Fitzgibbon A., Finocchio M., Blake A., Cook M., Moore R. (2013). Real-time human pose recognition in parts from single depth images. Commun. ACM.

[23] Ogawa A., Mita A., Georgoulas C., Bock T. (2016). A Face Recognition System for Automated Door Opening with parallel Health Status Validation Using the Kinect v2. Proceedings of the International Symposium on Automation and Robotics in Construction.

[24] Butkiewicz T. (2014). Low-cost coastal mapping using Kinect v2 time-of-flight cameras. Oceans-St. John’s, 2014.

[25] Yang L., Zhang L., Dong H., Alelaiwi A., El Saddik A. (2015). Evaluating and improving the depth accuracy of Kinect for Windows v2. IEEE Sens. J..

[26] Vicon. https://www.vicon.com.

[27] Vallabhajosula S., Yentes J.M., Momcilovic M., Blanke D.J., Stergiou N. (2012). Do lower-extremity joint dynamics change when stair negotiation is initiated with a self-selected comfortable gait speed?. Gait Posture.

[28] Vallabhajosula S., Yentes J.M., Stergiou N. (2012). Frontal joint dynamics when initiating stair ascent from a walk versus a stand. J. Biomech..

[29] Bogin B., Varela-Silva M.I. (2010). Leg length, body proportion, and health: A review with a note on beauty. Int. J. Environ. Res. Public Health.

[30] Eveleth P.B., Tanner J.M. (1976). Worldwide Variation in Human Growth.

[31] Plonus M. (2001). Electronics and Communications for Scientists and Engineers.

[32] Portney L., Watkins M. (1993). Foundations of clinical research: Application to practice. Stamford USA Appleton Lange.

[33] Koo T.K., Li M.Y. (2016). A guideline of selecting and reporting intraclass correlation coefficients for reliability research. J. Chiropr. Med..

